# LATE-LIFE SUICIDE: IDENTIFYING PREVENTION TARGETS AND MOBILIZING COMMUNITIES TO MANAGE RISK

**DOI:** 10.1093/geroni/igz038.3037

**Published:** 2019-11-08

**Authors:** Alexandria R Ebert, Emily S Bower, Yeates Conwell

**Affiliations:** 1 West Virginia University, Morgantown, United States; 2 VISN Center of Excellence for Suicide Prevention, Canandaigua, New York, United States; 3 University of Rochester School of Medicine, Rochester, New York, United States

## Abstract

Adults aged 70 and older have the highest suicide rates in most countries across the globe (World Health Organization, 2014). Prevalence may increase as the Baby Boomers transition into older adulthood (Phillips, 2014). Disease and disability increase risk for late-life suicide, thus identifying opportunities to intervene within the network of services utilized by older adults with illness and disability could reduce suicide risk. This symposium will explore the impact of disease and disability on late-life suicide risk, and present novel ideas for intervention and prevention. Emphasizing the conference theme, we will discuss prevention points spanning networks of care, including mental health and social services, and long-term care. Dr. Bower will frame the scope of the problem by presenting findings of age-stratified associations between physical illness and suicide attempt among older veterans using secondary data from a retrospective case-control study of veterans. Ms. Lutz will present findings on the relationship between disability and suicidal thoughts from a clinical trial of problem-solving therapy. Dr. Lane will discuss management and identification of suicide risk factors during the transition from independent living to long-term care through the lens of a case-series. Dr. Fullen will present preliminary findings from a novel, randomized controlled trial of a community-based suicide prevention training program for nutrition service volunteers. Dr. Yeates Conwell, Co-Director of the Center for the Study and Prevention of Suicide and Director of the Geriatric Psychiatry Program at the University of Rochester Medical Center, will serve as the discussant.

